# Cyclosporine A Causes Gingival Overgrowth by Promoting Entry into the S Phase at the G1/S Cell Cycle Checkpoint in Gingival Fibroblasts Exposed to Lipopolysaccharide

**DOI:** 10.3390/diseases12120322

**Published:** 2024-12-10

**Authors:** Reiri Takeuchi, Noriko Kuwahara, Yuta Amino, Sachiyo Hayashi, Chieko Taguchi, Itaru Suzuki, Haruka Suzuki, Teruaki Nagashima, Kazumune Arikawa, Yuichiro Okada, Takato Nomoto, Koichi Hiratsuka

**Affiliations:** 1 Department of Biochemistry and Molecular Biology, Nihon University School of Dentistry at Matsudo, Matsudo 271-8587, Chiba, Japan; kuwahara.noriko@nihon-u.ac.jp (N.K.); hiratsuka.koichi@nihon-u.ac.jp (K.H.); 2 Department of Oral Implantology, Nihon University School of Dentistry at Matsudo, Matsudo 271-8587, Chiba, Japan; amino.yuta@nihon-u.ac.jp; 3 Department of Special Needs Dentistry, Nihon University School of Dentistry at Matsudo, Matsudo 271-8587, Chiba, Japan; hayashi.sachiyo@nihon-u.ac.jp (S.H.); nomoto.takato@nihon-u.ac.jp (T.N.); 4 Department of Community Oral Health, Nihon University School of Dentistry at Matsudo, Matsudo 271-8587, Chiba, Japan; taguchi.chieko@nihon-u.ac.jp (C.T.); suzuki.itaru@nihon-u.ac.jp (I.S.); sakazume.haruka@nihon-u.ac.jp (H.S.); arikawa.kazumune@nihon-u.ac.jp (K.A.); 5 Department of Community Oral Health, Nihon University Graduate School of Dentistry at Matsudo, Matsudo 271-8587, Chiba, Japan; 6 Department of Histology, Nihon University School of Dentistry at Matsudo, Matsudo 271-8587, Chiba, Japan

**Keywords:** gingival overgrowth, cyclosporine A, gingival fibroblast, cell cycle, G1/S checkpoint, S-phase entry, G1-S transition

## Abstract

Objectives: Cyclosporine A promotes gingival fibrosis by enhancing the proliferation of gingival fibroblasts, leading to gingival overgrowth. The population of gingival fibroblasts is regulated by cell cycle machinery, which balances cell growth and inhibition. Cells that detect DNA damage pause at the G1/S checkpoint to repair the damage instead of progressing to the S phase. Previous studies have linked drug-induced gingival overgrowth to the response of fibroblasts to lipopolysaccharide (LPS) and cyclosporine A. This research investigates the effects of cyclosporine A on the G1/S checkpoint and its mediators in LPS-treated gingival fibroblasts to clarify the mechanisms behind cyclosporine-A-induced gingival overgrowth. Methods: Semi-confluent human gingival fibroblasts were treated with LPS or cyclosporine A in DMEM. Cell proliferation was evaluated by counting the total number of cells. The distribution of the cell cycle phases was analyzed using flow cytometry. Additionally, the expression levels of mRNAs and proteins related to cell cycle regulators were quantified by reverse-transcription quantitative PCR and Western blotting, respectively. Results: Cyclosporine A treatment significantly enhanced cell proliferation and the G1-S cell cycle transition. It increased the mRNA levels of *CDC25A* and *CYCLIN D* while decreasing those of *RB1*, *SMAD3*, and *SMAD4*. Additionally, it upregulated the protein levels of CDC25A, CYCLIN D, CDK4, CDK6, and pRB and downregulated the protein levels of SMAD3 and SMAD4. Conclusions: Gingival overgrowth induced by cyclosporine A could be attributed to these alterations.

## 1. Introduction

Cyclosporine A, an immunosuppressant commonly used after organ transplantation, is associated with gingival overgrowth. Studies have indicated that the prevalence of gingival overgrowth due to cyclosporine A ranges from 50% to 80% [1,2]. In 2022, organ transplants were conducted in 91 countries (157,494 cases) [3].

Drug-induced gingival overgrowth, also known as drug-induced gingival enlargement or hyperplasia, can result from not only cyclosporine A but also phenytoin (an anti-epileptic medication) and nifedipine (used for hypertension) [1,4,5,6]. The primary clinical feature of this condition is an enlargement of the gingiva [7], which can lead to a variety of complications, including difficulties with oral hygiene, impaired chewing function, painful mastication and eating, aesthetic concerns, and psychological distress [1,6,8,9,10].

Cyclosporine A promotes the development of gingival fibrosis through mechanisms such as enhanced proliferation, suppressed apoptosis of gingival fibroblasts, and collagen accumulation [2], which leads to gingival overgrowth [11,12]. Gingival fibroblasts are crucial for maintaining and repairing the connective tissues of the gingiva for the homeostasis of those tissues [13]. An in vitro model of gingival fibroblasts was employed to explore the pathogenic molecular actions responsible for cyclosporine-A-associated overgrowth in the gingiva, and research has focused on the effects of cyclosporine A on cultured gingival fibroblasts [12,14].

Changes in the fibroblast population within the gingival tissue are regulated by cell cycle machinery, which balances the proliferation and inhibition of cell growth [15,16]. The cell cycle consists of four distinct phases: the S phase to synthesize DNA; the M phase to divide the cell; the G1 phase to prepare for the S phase; and the G2 phase to prepare for the M phase. Checkpoint systems, including the G1/S and G2/M checkpoints, regulate the progression and halting of the cell cycle. The G1/S checkpoint specifically restricts cell cycle advancement; cells that detect DNA damage, for instance, do not transition to the S phase but instead halt at the G1/S checkpoint to facilitate damage [15,16,17].

Lipopolysaccharide (LPS), a component of the cell membranes of bacteria such as *Escherichia coli* and *Porphyromonas gingivalis*, helps maintain homeostasis in gingival tissues by inducing apoptosis and inflammation [18]. Additionally, LPS inhibits cell proliferation [19].

Drug-induced gingival overgrowth is linked to the response of gingival fibroblasts to lipopolysaccharide (LPS) and drugs such as cyclosporine A [18,20]. We hypothesized that cyclosporine A promotes fibroblast proliferation by increasing entry into the S phase at the G1/S checkpoint in gingival fibroblasts exposed to LPS, thereby contributing to gingival overgrowth. In this study, we examined the effects of cyclosporine A on the G1/S checkpoint and its mediators in LPS-treated gingival fibroblasts to elucidate the mechanisms underlying cyclosporine-A-induced gingival overgrowth. Our results indicated that cyclosporine A promotes entry into the S phase at the G1/S checkpoint by regulating the expression of mediator proteins involved in the transition to the S phase in LPS-treated gingival fibroblasts.

## 2. Materials and Methods

### 2.1. Cell Line and Culture

We performed this study using the methods described in previous reports [6,7,15,18,21]. Primary gingival fibroblasts used in this research were obtained from ScienCell^TM^ Research Laboratories (cat. no. 2620, San Diego, CA, USA). These gingival fibroblasts were derived from healthy donors, cryopreserved at passage one, and were delivered frozen. Cells were cultured according to manufacturer’s instructions in Dulbecco’s modified Eagle medium (High Glucose) with L-Glutamine and Phenol Red (D-MEM, FUJIFILM Wako Pure Chemical Corp., Osaka, Japan) supplemented with 10% fetal bovine serum, 50 units/mL of penicillin and 50 μg/mL of streptomycin (Gibco, Thermo Fisher Scientific, Inc., Waltham, MA, USA). Cells were grown under an atmosphere of 5% CO_2_/95% air and were maintained at 37 °C until they reached semi-confluence, after which they were routinely passaged using 0.05 *w*/*v*% Trypsin-0.53 mmol/L EDTA-4Na Solution with Phenol Red (FUJIFILM Wako Pure Chemical Corp.). Experiments were performed using cells between passages 6 and 9. Cyclosporine A (cat. no. 031-24931) and LPS (cat. code: tlrl-pglps) were purchased from FUJIFILM Wako Pure Chemical Corp. (Osaka, Japan) and InvivoGen (San Diego, CA, USA), respectively. The concentration of cyclosporine A was determined due to the results of a previous study [22], outlined as follows: cell proliferation in normal gingival fibroblasts treated with cyclosporine A (200 ng/mL) significantly increased compared to that in untreated control fibroblasts. The concentration of LPS was determined due to the following results of a previous study [23]: LPS concentrations ranging from 0.01 to 10 μg/mL inhibited the proliferation of human gingival fibroblasts. Specifically, treatment with 1 μg/mL of LPS significantly activated p38, a key regulator of cell cycle arrest.

### 2.2. Evaluation of Cell Proliferation

We counted the total number of cells to evaluate cell proliferation with Trypan Blue dye exclusion using a blood corpuscle counting chamber (Burker-Turk deep 1/10 mm, ERMA Inc., Tokyo, Japan) and observed the cellular morphology using an optical microscope (Nikon TE300 Inverted Tissue Culture Microscope, Tokyo, Japan; magnification, 40×). After semi-confluent cells were cultured in D-MEM with 1% fetal bovine serum for 24 h to arrest cell growth, cells were treated with or without 200 ng/mL of cyclosporine A with or without 1 μg/mL of LPS in D-MEM containing 5% fetal bovine serum for 24 or 48 h. After arrest, cells were continuously cultured in fresh DMEM containing 5% serum for 24 h as a negative control. The methods used in this study were based on a previously published report [15].

### 2.3. Analysis of Cell Cycle Phase Distribution

The distribution of cell cycle phases was analyzed using flow cytometry with the CycleTEST™ Plus DNA Reagent Kit (Becton Dickinson and Company, Franklin Lakes, NJ, USA). Semi-confluent cells were cultured in D-MEM supplemented with 1% fetal bovine serum for 24 h to induce cell cycle arrest at the G0/G1 phase. Subsequently, the cells were treated with or without 200 ng/mL of cyclosporine A in D-MEM containing 5% fetal bovine serum and 1 μg/mL of LPS for an additional 24 h. Following treatment, cells were harvested via trypsinization, washed three times with buffer solution, and then processed with trypsin buffer (Solution A), trypsin inhibitor, RNase buffer (Solution B), and propidium iodide staining solution (Solution C) according to the manufacturer’s guidelines. Each sample (2 × 10^5^ cells) was analyzed using a FACSCalibur™ flow cytometer (BD Biosciences, Franklin Lakes, NJ, USA), and the percentages of cells in the G0/G1, S, and G2/M phases of the cell cycle were calculated using BD CellQuest Pro Software (version 3.1, BD Biosciences). The methodologies employed in this study were adapted from the previously published literature [6,7,15,21].

### 2.4. Analysis of mRNA Expression

RNA isolation and quantitative reverse transcription PCR (RT-qPCR) were performed to analyze mRNA expression levels. After culturing the cells until they reached semi-confluence, they were placed in D-MEM with 1% fetal bovine serum for 24 h. The cells were then treated with or without 200 ng/mL of cyclosporine A in D-MEM containing 5% fetal bovine serum and 1 μg/mL of LPS for 12 h. Total RNA was immediately extracted from the cells using the RNeasy Mini Kit (QIAGEN, Tokyo, Japan). The concentration and purity of each total RNA sample were assessed using standard spectrophotometric methods. Reverse transcription was performed using one microgram of total RNA from each sample with the PrimeScript™ RT Reagent Kit (TAKARA BIO Inc., Shiga, Japan). The resulting cDNAs were analyzed via qPCR using the Eco™ Real-Time PCR System (Illumina, Inc., San Diego, CA, USA) and KAPA SYBR^®^ FAST qPCR Master Mix Kit (KAPA BIOSYSTEMS Inc., Wilmington, MA, USA). The qPCR thermocycling conditions were as follows: enzyme activation at 95 °C for 30 s, followed by 45 cycles of denaturation at 95 °C for 5 s, and annealing and extension at 60 °C for 20 s. The Perfect Real Time Support System (TAKARA BIO Inc.) was utilized to synthesize the PCR primers for the following genes: BMI1 proto-oncogene polycomb ring finger (*BMI1*); cell division cycle 25A (*CDC25A*); cyclin-dependent kinase 4 (*CDK4*); cyclin-dependent kinase 6 (*CDK6*); D-type cyclin (*CYCLIN D*); glycogen synthase kinase 3 beta (*GSK3B*); MYC proto-oncogene bHLH transcription factor (*MYC*); cyclin-dependent kinase inhibitor 2B (*P15*); cyclin-dependent kinase inhibitor 2A (*P16*); cyclin-dependent kinase inhibitor 2C (*P18*); cyclin-dependent kinase inhibitor p19INK4d (*P19*); RB transcriptional corepressor 1 (*RB1*); SMAD family member 3 (*SMAD3*); SMAD family member 4 (*SMAD4*); and glyceraldehyde-3-phosphate dehydrogenase (*GAPDH*). These primers were synthesized using Custom DNA Oligos (Merck KGaA, Darmstadt, Germany) based on the nucleotide database from the National Center for Biotechnology Information (National Library of Medicine, National Institutes of Health, Bethesda, MD, USA) and Primer3Plus. The sequences of the primers are provided in Table 1. Relative quantification was performed using the 2^−∆∆Cq^ method [24]. After normalizing to *GAPDH*, the RNA ratios between cyclosporine-A-treated and untreated control cultures were calculated. The methodologies employed in this study were adapted from the previously published literature [6,7].

### 2.5. Analysis of Protein Expression

Protein expression levels were assessed using Western blotting. Cells were cultured until semi-confluence and then incubated in D-MEM with 1% fetal bovine serum for 24 h. Subsequently, the cells were treated with or without 200 ng/mL of cyclosporine A in D-MEM containing 5% fetal bovine serum and 1 μg/mL of LPS for another 24 h. After treatment, the cells were washed with PBS (37 °C) and lysed using β-ME Sample Treatment for Tris SDS (COSMO BIO Co., Ltd., Tokyo, Japan). Protein concentrations were determined using a TaKaRa Bradford Protein Assay Kit (TAKARA BIO Inc.). Subsequently, a total of 10 μg of protein per lane was separated by SDS-PAGE using a running buffer solution (NACALAI TESQUE, Inc., Kyoto, Japan) and transferred to PVDF membranes. The membranes were blocked for 30 min at room temperature with Bullet Blocking One for Western blotting (NACALAI TESQUE) and incubated with primary antibodies against CDC25A, CYCLIN D, CDK4, CDK6, pRB, RB, SMAD3, SMAD4, and β-Actin for 1 h at room temperature. After three 5-minute washes with Tris-buffered saline containing 0.05% detergent (NACALAI TESQUE), the membranes were incubated with secondary antibodies for 45 min at room temperature. Both primary and secondary antibodies were diluted to 1:1000 and 1:10,000, respectively, in Can Get Signal^®^ Immunoreaction Enhancer Solution (TOYOBO Co., Ltd., Osaka, Japan). After washing, the blots were analyzed using a Chemi-Lumi One Super (NACALAI TESQUE) and a ChemiDoc™ MP Imaging System (Bio-Rad Laboratories, Inc., Hercules, CA, USA). The densities of the Western blot bands were quantified using ImageJ (version 1.53t; Java 1.8.0_345 [64-bit]). The primary antibodies used included pRB1 (cat. no. #9308), SMAD3 (cat. no. #9523), SMAD4 (cat. no. #46535), and β-Actin (cat. no. #4967), along with anti-rabbit HRP-conjugated IgG and RB (anti-mouse IgG, HRP-linked antibody; cat. no. #9309), all sourced from Cell Signaling Technology, Inc. (Danvers, MA, USA). The rabbit anti-CDC25A antibody (cat. no. 55031-1-AP) and mouse antibodies against CDK4 (cat. no. 66950-1-Ig), CDK6 (cat. no. 66278-1-Ig), and CYCLIN D (cat. no. 60186-1-Ig) were obtained from Proteintech Group Inc. (Rosemont, IL, USA). The secondary HRP-linked antibodies against rabbit IgG (cat. no. #65-6120) and mouse IgG (cat. no. NA931V) were purchased from Invitrogen (Thermo Fisher Scientific Inc., Waltham, MA, USA) and GE Healthcare UK Limited (Amersham Place, Buckinghamshire, UK). The methodologies employed in this study were adapted from previously published reports [6,7].

### 2.6. Statistical Analysis

All data are presented as the mean ± standard error of the mean (SEM). Statistical analyses were conducted using Welch’s *t*-test. Furthermore, we employed analysis of variance (ANOVA) to assess differences between groups. Following the ANOVA results, Welch’s *t*-test with Bonferroni’s correction was applied as needed to identify specific differences among the groups. Statistical significance was set at *p* < 0.05.

## 3. Results

### 3.1. The Proliferation of Gingival Fibroblasts in the Presence of LPS and Cyclosporine A

**Figure 1:**
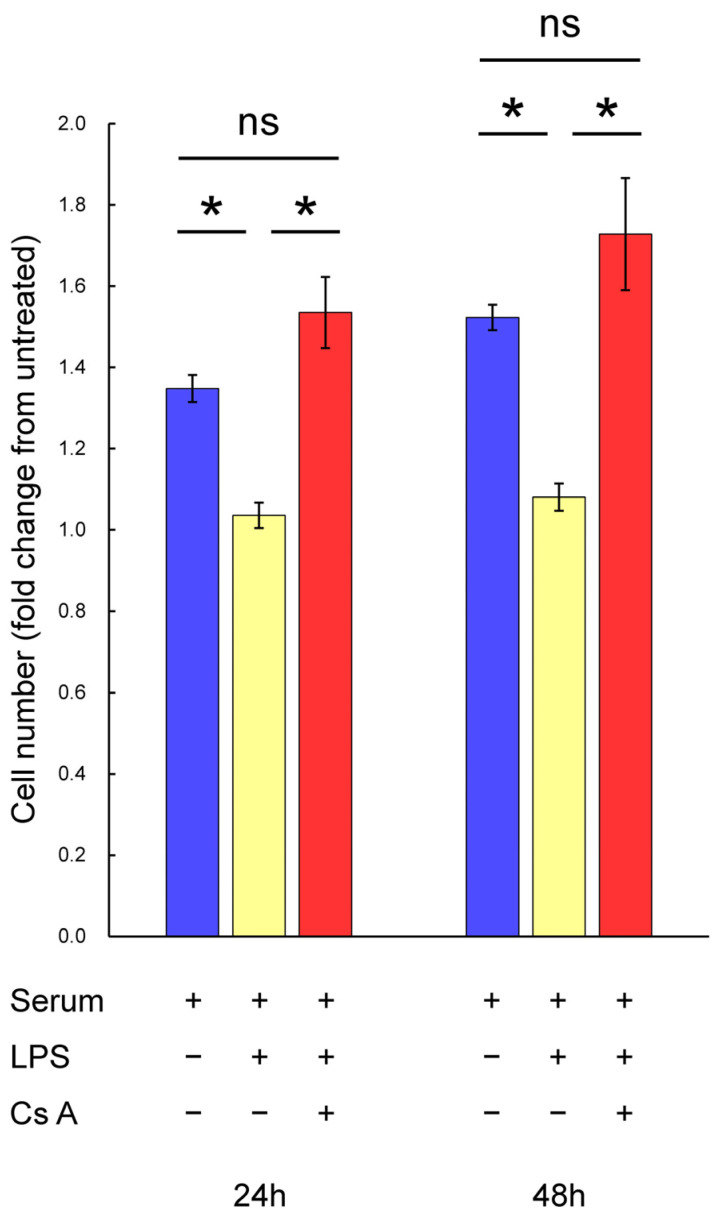
The proliferation of gingival fibroblasts stimulated with or without LPS or cyclosporine A in the culture with D-MEM plus serum. After semi-confluent cells were cultured in D-MEM containing 1% serum for 24 h, cells were treated with or without 200 ng/mL of cyclosporine A or 1 μg/mL of LPS in D-MEM containing 5% serum for 24 or 48 h. The total number of cells was measured with Trypan Blue dye exclusion using a blood corpuscle counting chamber and an optical microscope. The fold change from the negative control was calculated. Data are presented as means ± SEM. Welch’s *t*-test with Bonferroni’s correction was used for statistics. * *p* < 0.05 indicates a statistically significant difference. *n* = 4. Cs A, cyclosporine A.

Gingival fibroblasts were stimulated with or without LPS alone or with LPS plus cyclosporine A in DMEM containing serum for 24 and 48 h, and fibroblast proliferation was assessed. The results are shown in Figure 1. Treatment with serum for 24 h increased the number of gingival fibroblasts by 1.4-fold, and for 48 h, it increased by 1.5-fold compared to untreated cells. However, stimulation with LPS significantly decreased the number of gingival fibroblasts treated with serum at 24 h (0.8-fold) or 48 h (0.7-fold) compared to that in serum-only treated cells. Stimulation with cyclosporine A significantly increased the number of gingival fibroblasts treated with serum and LPS at 24 h (1.5-fold) or 48 h (1.6-fold) compared to serum–LPS-treated cells. The number of gingival fibroblasts observed when treated with serum alone or serum–LPS–cyclosporine A was similar.

### 3.2. Effect of Cyclosporine A on Cell Cycle Distribution in Gingival Fibroblasts Exposed to LPS

**Figure 2:**
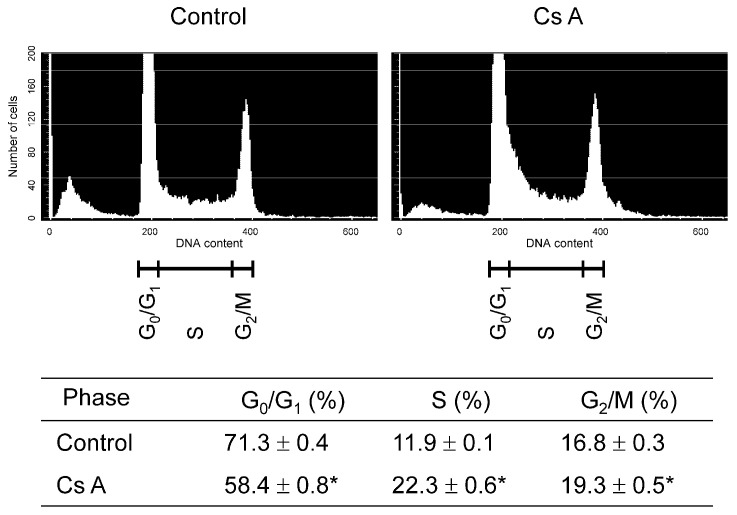
The cell cycle phase distribution of gingival fibroblasts cultured with or without cyclosporine A. After semi-confluent cells were cultured in D-MEM with 1% serum for 24 h, cells were treated with or without (control) 200 ng/mL of cyclosporine A in D-MEM containing 5% serum and 1 μg/mL of LPS for 24 h, and subjected to flow cytometric analysis. A representative histogram from four independent experiments is shown. Detailed values of cell cycle parameters are shown in the table at the bottom. Data are presented as means ± SEM. * *p* < 0.05 compared with control using Welch’s *t*-test (*n* = 4). Cs A, cyclosporine A.

Gingival fibroblasts were stimulated with or without cyclosporine A in the presence of LPS, and the distribution of the cell cycle phases (G0/G1, S, and G2/M) was analyzed. The results are shown in Figure 2. The number of cells in the G0/G1 phase or in the S and G2/M phases significantly decreased or increased after stimulation with LPS plus cyclosporine A, respectively, compared to the LPS-stimulated control.

### 3.3. Effect of Cyclosporine A on mRNA Expression in Gingival Fibroblasts Exposed to LPS

**Figure 3:**
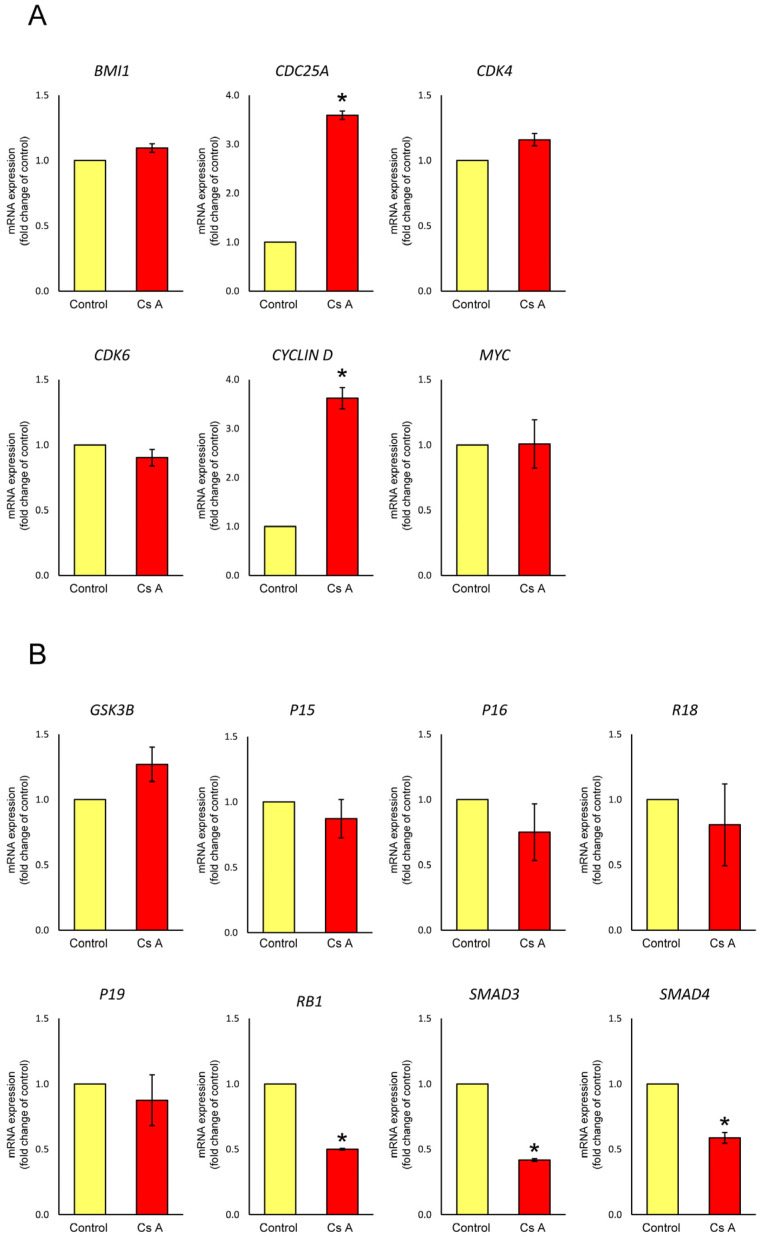
mRNA expression levels of mediators for G1-S cell cycle transition in gingival fibroblasts cultured in the presence or absence of cyclosporine A. After semi-confluent cells were cultured in D-MEM with 1% serum for 24 h, cells were treated with or without (control) 200 ng/mL of cyclosporine A in D-MEM containing 5% serum and 1 μg/mL of LPS for 12 h, after which, quantitative reverse-transcription PCR analysis was performed. Relative quantification was performed using the 2^−∆∆Cq^ method. Data were normalized to *GAPDH*. The ratios of RNAs in cyclosporine A vs. control cultures were determined. Data are presented as means ± SEM. * *p* < 0.05 compared with control using Welch’s *t*-test (*n* = 3): (**A**) G1-S transition-promoter genes (*BMI1*, BMI1 proto-oncogene polycomb ring finger; *CDC25A*, cell division cycle 25A; *CDK4*, cyclin-dependent kinase 4; *CDK6*, cyclin-dependent kinase 6; *CYCLIN D*, D-type cyclin; *MYC*, MYC proto-oncogene, bHLH transcription factor); (**B**) G1-S transition-inhibitor genes (*GSK3B*, glycogen synthase kinase 3 beta; *P15*, cyclin-dependent kinase inhibitor 2B (CDKN2B); *P16*, cyclin-dependent kinase inhibitor 2A (CDKN2A); *P18*, cyclin-dependent kinase inhibitor 2C (CDKN2C); *P19*, CDK inhibitor p19INK4d; *RB1*, RB transcriptional corepressor 1; *SMAD3*, SMAD family member 3; *SMAD4*, SMAD family member 4). Cs A, cyclosporine A.

The effects of cyclosporine A stimulation on the mRNA expression levels of regulators of the G1-S cell cycle transition (*BMI1*, *CDC25A*, *CDK4*, *CDK6*, *CYCLIN D*, *MYC*, *GSK3B*, *P15*, *P16*, *P18*, *P19*, *RB1*, *SMAD3*, and *SMAD4*) were analyzed using qPCR in gingival fibroblasts exposed to LPS. The results are shown in Figure 3. The mRNA expression levels of *CDC25A* (3.6-fold) and *CYCLIN D* (3.6-fold) were significantly upregulated. In contrast, the mRNA expression levels of *RB1* (0.5-fold), *SMAD3* (0.4-fold), and *SMAD4* (0.6-fold) were significantly downregulated in gingival fibroblasts stimulated with LPS plus cyclosporine A compared to those in the LPS-stimulated control. In addition, the mRNA expression levels of *CDK4* (1.2-fold) and *GSK3B* (1.3-fold) were slightly increased, whereas the mRNA expression levels of *P16* (0.7-fold), and *P18* (0.8-fold) were slightly decreased in cells stimulated with LPS plus cyclosporine A, but not significantly. The mRNA expression levels of *BMI1*, *CDK6*, *MYC*, *P15*, and *P19* in cells stimulated with LPS and cyclosporine A were similar to those in the LPS-stimulated control cells.

### 3.4. Effect of Cyclosporine A on Protein Expression in Gingival Fibroblasts Exposed to LPS

**Figure 4:**
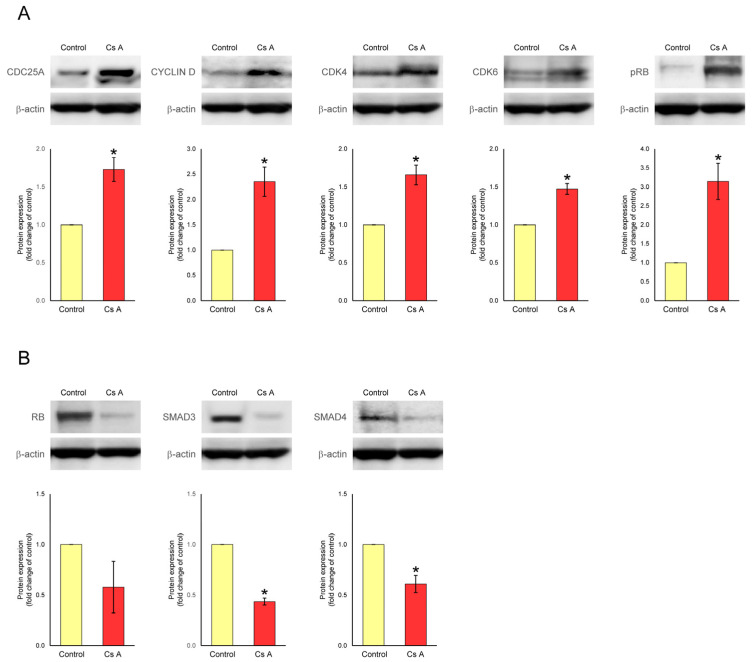
Protein expression levels of mediators for G1-S cell cycle transition in gingival fibroblasts cultured in the presence or absence of cyclosporine A. After semi-confluent cells were cultured in D-MEM with 1% serum for 24 h, cells were treated with or without (control) 200 ng/mL of cyclosporine A in D-MEM containing 5% serum and 1 μg/mL of LPS for 24 h, after which, Western blot analysis was performed. The fold change from control was calculated. Representative band images from three independent experiments are shown. Data are presented as means ± SEM. * *p* < 0.05 compared with control using Welch’s *t*-test (*n* = 3); (**A**) G1-S transition-promoter proteins (CDC25A, cell division cycle 25A; CYCLIN D, D-type cyclin; CDK4, cyclin-dependent kinase 4; CDK6, cyclin-dependent kinase 6; pRB, phospho-retinoblastoma protein); (**B**) G1-S transition-inhibitor proteins (RB, retinoblastoma protein; SMAD3, SMAD family member 3; SMAD4, SMAD family member 4). Cs A, cyclosporine A. Original images of Figure 4 are available in the Supplementary File.

The effects of cyclosporine A stimulation on the expression of regulatory proteins for the G1-S cell cycle transition (CDC25A, CYCLIN D, CDK4, CDK6, pRB, RB, SMAD3, and SMAD4) in gingival fibroblasts exposed to LPS were analyzed using Western blotting. The results are shown in Figure 4. The protein expression levels of CDC25A (1.7-fold), CYCLIN D (2.4-fold), CDK4 (1.7-fold), CDK6 (1.5-fold), and pRB (3.1-fold) significantly increased, whereas the protein expression levels of SMAD3 (0.4-fold) and SMAD4 (0.6-fold) significantly decreased in gingival fibroblasts stimulated with LPS plus cyclosporine A compared to the protein expression levels observed in the LPS-stimulated control cells. Stimulation with LPS plus cyclosporine A also slightly decreased RB mRNA expression (0.6-fold) levels but not significantly.

## 4. Discussion

This study aimed to elucidate the mechanism underlying gingival overgrowth caused by cyclosporine A. We investigated the impact of cyclosporine A on the G1/S cell cycle checkpoint and its mediators in gingival fibroblasts exposed to LPS, using a technically replicative approach. Our findings revealed that cyclosporine A modulates the expression of mediator proteins that enable passage through the G1/S checkpoint in LPS-responsive gingival fibroblasts, resulting in increased entry into the S phase.

Gingival overgrowth occurs due to the adverse effects of drugs like cyclosporine A through enhanced proliferation and diminished programmed cell death, known as “apoptosis”, in gingival fibroblasts [14,22]. Additionally, inflammatory responses in the gingiva play a significant role in the initiation and progression of drug-induced gingival overgrowth [25]. LPS released from *Porphyromonas gingivalis* triggers inflammation in the gingival tissue [18], whereas the presence of LPS reduces the proliferation of gingival fibroblasts [19]. Cell proliferation is controlled by phase transitions and arrest at checkpoints within the cell cycle. We stimulated gingival fibroblasts with serum alone, serum plus LPS, and serum plus LPS plus cyclosporine A and evaluated the effects of cyclosporine A on cell proliferation by counting the total number of cells. These results indicated that cyclosporine A increased the number of gingival fibroblasts against the reduced proliferation caused by LPS. Additionally, treatment with serum and cyclosporine A produced results similar to those obtained using serum alone.

We further evaluated the effects of cyclosporine A on the cell cycle of gingival fibroblasts exposed to LPS by flow cytometry, and the results showed that cyclosporine A increased the number of cells in the S and G2/M phases while decreasing the number of cells in the G0/G1 phase. Treatment with cyclosporine A induced a G1-S cell cycle transition in gingival fibroblasts in the presence of LPS, which might result in gingival overgrowth. The effects of cyclosporine A on cell proliferation and the cell cycle have been previously studied in various human cell types, yielding the following findings: Cyclosporine A activates the extracellular signal-regulated kinase (ERK1/2) signaling pathway and enhances the proliferation of HepG2 (human hepatocellular carcinoma-derived cell line) proliferation [26]. Cyclosporine A also promotes the growth of A549 cells (a human non-small-cell lung cancer cell line) [27] and human umbilical vein endothelial cells [28]. Cyclosporine A promoted the transition to the S phase followed by the G2/M phase from the G0/G1 phase in the cell cycle in Madin–Darby canine kidney (MDCK) cells (a renal tubular epithelial cell line derived from the distal tubular segment of the nephron) [29]. Treatment with cyclosporine A causes gingival overgrowth via enhancement of progression to the G2/M phase from the G0/G1 and S phases of the cell cycle in gingival fibroblasts [30]. Additionally, cyclosporine A induced an increase in the number of cells in the S and G2/M phases of the cell cycle in primary cultures of rat hepatocytes, resulting in increased cell proliferation [31]. In the present study, using human gingival fibroblasts, our results provide new observations that are more advanced than previous findings.

Cell division is controlled by cell cycle machinery. Regulation by the cell cycle also provides an opportunity to repair the damage and block defective transmission to daughter cells [32]. The essential components controlling the cell cycle are cyclins, which bind to their catalytic partners, cyclin-dependent kinases (CDKs). This interaction allows CDKs to gain substrate specificity [33,34,35,36,37]. The cell cycle consists of four distinct phases: the gap 1 phase (G1), the DNA synthetic phase (S), the gap 2 phase (G2), and the mitotic phase (M). Additionally, the cell cycle features checkpoint systems, including the G1/S checkpoint located in the G1 phase. At this checkpoint, cells select a specific pathway for progression based on the mitogenic conditions they encounter. This choice can either lead to the activation of a program that promotes cell division or result in cells entering a quiescent G0 [33,34,35,36,37].

At the molecular level, the presence of growth-promoting factors stimulates cells to upregulate D-type cyclins (D1, D2, and D3), which in turn activate CDK4 and CDK6 [33,34,35,36,37,38]. This activation facilitates the transition of cells from the G1 phase to the S phase of the cell cycle, thereby initiating DNA synthesis. CDK4/CDK6 complexes bind to CYCLIN D and, subsequently, phosphorylate the retinoblastoma protein RB1 [39]. In its hypophosphorylated state, RB1 typically binds to E2F transcription factors, inhibiting their activity and preventing cells from entering the S phase [39]; however, when RB1 is phosphorylated by CYCLIN D-CDK4/CDK6 complexes, E2F transcription factors are released, leading to entry into the S phase [39,40,41]. Cell Division Cycle-25A (CDC25A) is crucial for facilitating the transition between the G1 and S phases of the cell cycle by activating CDK4/6. Cell cycle checkpoints regulate CDC25A levels. Overexpression of CDC25A disrupted the normal progression of the G1-S transition. Notably, CDC25A is abundantly expressed during the late G1 phase (at the G1/S checkpoint), promoting the progression of cells into the S phase [42]. The MYC proto-oncogene bHLH transcription factor (MYC), also known as c-Myc, plays a pivotal role in stimulating cell cycle progression. MYC specifically targets key cell cycle regulators, including CYCLIN D, CYCLIN E, CDK4/6, CDC25A, and E2F. Upon activation of CYCLIN D, CDK4/6, and CDC25A, MYC effectively drives the transition from the G1 phase to the S phase, thereby promoting cellular proliferation [43].

At this checkpoint, cell-cycle progression is either delayed or halted in response to DNA damage. This pause allows the necessary time for repair mechanisms to operate before the cell proceeds to subsequent phases of the cycle. During the G1/S checkpoint, the INK4 family of cyclin-dependent kinase inhibitors, including P16INK4a, P15INK4b, P18INK4c, and P19INK4d, play a critical role in halting cell cycle progression by binding directly to CYCLIN D/CDK4/6 complexes and inhibiting their activity [44,45]. Additionally, Glycogen synthase kinase-3β (GSK-3β)—a serine/threonine protein kinase —is essential for preventing the G1-S transition of the cell cycle as it inactivates CYCLIN D1, thereby inhibiting cellular progression [46]. B-cell-specific Moloney murine leukemia virus integration site 1 (BMI1) plays a crucial role in advancing the cell cycle from the G1 phase to the S phase by boosting CDK4/6 activity through the suppression of P16Ink4a, which in turn accelerates cell proliferation [47]. SMADs are a family of proteins that transmit extracellular signals directly into the nucleus. In mammalian cells, there are eight distinct SMADs (SMAD1-8). Among them, SMAD4 is the only human Co-SMAD that partners with R-SMADs such as SMAD3 to recruit co-regulators to the signaling complex. The interaction between SMAD4 and SMAD3 forms a complex that plays a critical role at the G1/S checkpoint, maintaining cells in the G1 phase and ultimately resulting in cell cycle arrest [48]. Additionally, the activation of SMAD3 results in a reduction in CDC25A expression, which subsequently leads to cell cycle arrest [49].

**Figure 5:**
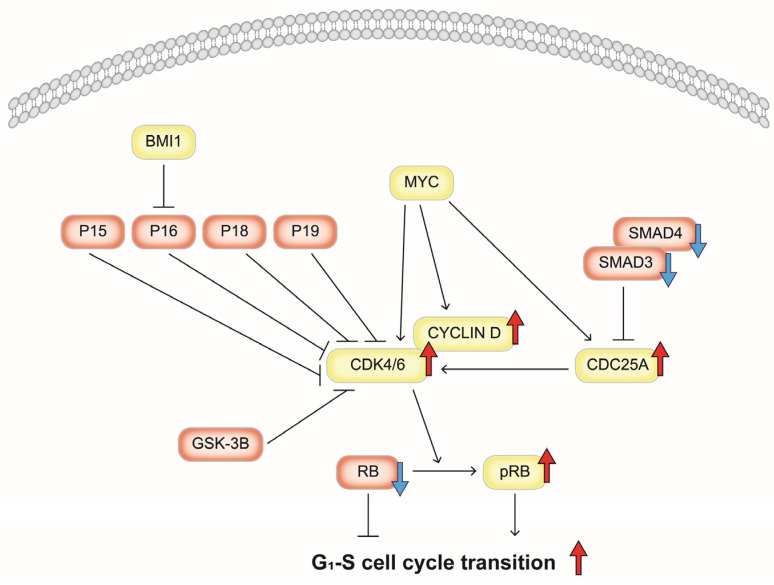
This schematic illustrates the process by which cyclosporine A promotes entry into the S phase at the G1/S cell cycle checkpoint in gingival fibroblasts. Cyclosporine A triggers the downregulation of SMAD3 and SMAD4, increasing CDC25A levels. Furthermore, it stimulates the upregulation of CYCLIN D, CDK4, and CDK6, which leads to elevated levels of phosphorylated RB. This cascade ultimately facilitates the transition into the S phase from the G1 phase. Additionally, cyclosporine A contributes to the downregulation of RB. The light yellow rectangles represent molecules involved in signaling that promotes the G1/S transition, whereas the light red rectangles indicate those that inhibit this transition, as analyzed in this study. The large blue arrows signify downregulation whereas the large red arrows indicate upregulation, both as a result of cyclosporine A treatment. BMI1, BMI1 proto-oncogene polycomb ring finger; CDC25A, cell division cycle 25A; CDK4, cyclin-dependent kinase 4; CDK6, cyclin-dependent kinase 6; CYCLIN D, D-type cyclin; GSK-3B, glycogen synthase kinase 3 beta; MYC, MYC proto-oncogene, bHLH transcription factor; pRB, phosphorylated RB transcriptional corepressor; P15, cyclin-dependent kinase inhibitor 2B (CDKN2B); P16, cyclin-dependent kinase inhibitor 2A (CDKN2A); P18, cyclin-dependent kinase inhibitor 2C (CDKN2C); P19, CDK inhibitor p19INK4d; RB, RB transcriptional corepressor; SMAD3, SMAD family member 3; SMAD4, SMAD family member 4.

To explore the effect of cyclosporine A on the regulators involved in cell cycle progression and arrest at the G1/S checkpoint, we analyzed the mRNA expression levels of various genes associated with the G1-S transition. This included inducers such as *BMI1*, *CDC25A*, *CDK4*, *CDK6*, *CYCLIN D*, and *MYC*, as well as inhibitors like *GSK-3β*, *P15*, *P16*, *P18*, *P19*, *RB1*, *SMAD3*, and *SMAD4*. Additionally, we assessed the protein expression levels of CDC25A, CYCLIN D, CDK4, CDK6, phospho-RB, RB, SMAD3, and SMAD4 in gingival fibroblasts treated with LPS. Our findings indicated that cyclosporine A treatment led to an increase in the mRNA expression levels of *CDC25A* and *CYCLIN D* while simultaneously decreasing the mRNA levels of *RB1*, *SMAD3,* and *SMAD4*. Additionally, we observed an increase in the protein expression levels of CDC25A, CYCLIN D, CDK4, CDK6, and phosphorylated RB (pRB) along with a reduction in the protein levels of SMAD3 and SMAD4 in LPS-treated gingival fibroblasts. Based on these findings, cyclosporine A may enhance phospho-RB by increasing the levels of CYCLIN D, CDK4, and CDK6 while upregulating CDC25A and downregulating SMAD3 and SMAD4. This alteration could facilitate the transition from G1 to S phase by disrupting G1 arrest at the G1/S checkpoint in LPS-treated gingival fibroblasts. Gingival overgrowth induced by cyclosporine A may be attributed to this mechanism (shown schematically in Figure 5).

In a previous study, the effect of cyclosporine A on cell cycle regulators was examined in various mammalian cell types. Treatment with cyclosporine A led to increased RB1 phosphorylation by enhancing the expression of CDK4, CYCLIN D1, and RB proteins in human gingival fibroblasts [50]. Additionally, the proliferation of A431 human epidermoid carcinoma (CRL-2592) cells has been linked to elevated levels of the CYCLIN D1 protein [51]. In human gingival fibroblasts from patients experiencing gingival overgrowth due to cyclosporine A, the mRNA expression of CYCLIN B1 was higher than that in normal gingival fibroblasts from healthy individuals [30]. Cyclosporine A also increased the mRNA and protein levels of CYCLIN D1, decreased P27 mRNA expression, and upregulated phospho-AKT/AKT protein levels, which helped bypass cell cycle arrest in the G1 and G2 phases owing to reduced P27 protein levels in A549 cells [27]. Furthermore, in primary cultures of rat hepatocytes treated with cyclosporine A, there was an increase in the protein levels of CYCLIN D1, CYCLIN E1, and PCNA (proliferating cell nuclear antigen), whereas P27 levels remained unchanged [31]. Our results not only elucidate the mechanisms underlying cyclosporine-A-induced gingival overgrowth but also offer new insights into its impact on the cell cycle in human gingival fibroblasts, building on previous research.

Cyclosporine-A-induced gingival overgrowth may be alleviated by inducing cell cycle arrest through the downregulation of CDC25A, CDK4/6, CYCLIN D, and pRB, along with the enhancement of SMAD3, SMAD4, and RB in gingival fibroblasts. Our previous research demonstrated that glycyrrhetinic acid, a natural compound derived from licorice and widely used in traditional Chinese medicine, is effective in treating drug-induced gingival overgrowth [7,15]. Notably, glycyrrhetinic acid has also been shown to induce G1-phase cell cycle arrest in human non-small-cell lung cancer cells by downregulating CDK4/6, CYCLIN D, and pRB [52]. Future research will explore the effects of glycyrrhetinic acid on these cell cycle regulators in gingival fibroblasts treated with cyclosporine A.

The progression and arrest of the cell cycle at the G1/S checkpoint are controlled by CYCLIN E-CDK2 complexes, members of the CIP/KIP family (including P21CIP1, P27KIP1, and P57KIP2), as well as key proteins such as P53, ATM (ataxia-telangiectasia mutated), ATR (ataxia-telangiectasia mutated, and rad3 related), CHK1, and CHK2, along with CYCLIN D-CDK4/6 complexes and its regulators [17,53,54]. Phosphorylation of RB1 by CYCLIN D-CDK4/CDK6 complexes results in the release of E2F transcription factors, which in turn enhance the transcription of E2F target genes, specifically CYCLIN E1 and CYCLIN E2 [39,40,41]. E-type cyclins subsequently associate with and activate CDK2, resulting in the hyperphosphorylation of RB1 and phosphorylation of various other proteins. This series of events collectively pushed the cell into an irreversible commitment to the S phase [39,40,41]. The activity of CYCLIN E-CDK2 complexes is modulated by the inhibitors P21CIP1, P27KIP1, and P57KIP2, which directly bind to the complex and prevent its catalytic function [55]. CYCLIN E-CDK2 can phosphorylate its inhibitor P27KIP1, marking it for degradation, which in turn enhances CDK2 kinase activity [55]. ATM and ATR play a role in activating P53, which in turn leads to the upregulation of P21 [53]. Furthermore, the expression of P21 is tightly regulated by various factors, including breast cancer susceptibility gene 1 (BRCA1), double homeobox 4 (Dux4), and Promyelotic zinc finger (PLZF) [56]. The progression of the cell cycle during the G1 phase is primarily regulated by the PI3K/AKT signaling pathway. Upon stimulation by growth factors, AKT is activated via PI3K, which subsequently promotes the activation of the CYCLIN D-CDK4 through the regulation of P21CIP1, P27KIP1, GSK-3B, and C-FOS, allowing the cell cycle to transition from the G1 phase to the S phase [57]. Furthermore, cell progression is supported by JNK1, JNK2, and JNK3. For example, fibroblast proliferation can be inhibited by G2/M cell cycle arrest when JNK2 is inhibited [58,59]. Future studies should focus on elucidating the effects of cyclosporine A on these regulatory factors in gingival fibroblasts.

## 5. Conclusions

Gingival overgrowth induced by cyclosporine A may be attributed to the following mechanism: cyclosporine A enhances phospho-RB by increasing CYCLIN D, CDK4, and CDK6 while also upregulating CDC25A and downregulating SMAD3 and SMAD4, which may facilitate the transition from the G1 phase to the S phase at the G1/S checkpoint in LPS-treated gingival fibroblasts.

## Figures and Tables

**Table 1:** Primers used for quantitative reverse-transcription PCR.

Gene Symbol	Sequence (5′-3′)	Product Size, bp	GenBank Accession No.
*BMI1*	F: GCTCATCCTTCTGCTGATGC	224	OP680450.1
	R: TGCATCACAGTCATTGCTGC		
*CDC25A*	F: CAAGGGTGCAGTGAACTTGC	95	NM_001789.3
	R: CAACAATGACACGCTTGCCA		
*CDK4*	F: ACTCTGAAGCCGACCAGTTG	84	NM_000075.4
	R: GCAGGGATACATCTCGAGGC		
*CDK6*	F: GTGTGCACAGTGTCACGAAC	194	NM_001259.8
	R: AGATCGCGATGCACTACTCG		
*CYCLIN D*	F: AGCTGTGCATCTACACCGAC	160	NM_053056.3
	R: TGTTTGTTCTCCTCCGCCTC		
*GSK3B*	F: GAAGTGCAAAGCAGCTGGTC	133	NM_002093.4
	R: ACACAGCCAGCAGACCATAC		
*MYC*	F: CATCAGCACAACTACGCAGC	169	NM_002467.6
	R: CGTTGTGTGTTCGCCTCTTG		
*P15*	F: AGCCCAGGTCTCCTAGGAAG	214	NM_004936.4
	R: CGCACCTTCTCCACTAGTCC		
*P16*	F: ACCAGAGGCAGTAACCATGC	93	NM_000077.5
	R: CCGAGGTTTCTCAGAGCCTC		
*P18*	F: GCATCACTCTCCTTCCTCGG	288	NM_001262.3
	R: TTCGGTGGCCCTCAAGTTAC		
*P19*	F: CCAATGTCCAGGACACCTCC	166	U40343.1
	R: ACAGCAGTGTGACCCTCTTG		
*RB1*	F: ATGTCTTTATTGGCGTGCGC	123	NM_000321.3
	R: AGAGCCATGCAAGGGATTCC		
*SMAD3*	F: TCTGAGAGGGCCAAATGCTG	86	NM_005902.4
	R: CAGGGGGCTTCCTGTGTAAG		
*SMAD4*	F: TGGTAGAGGCCAGCTTTGTG	103	NM_005359.6
	R: TCAATCCAAGCCCGTGAGTC		
*GAPDH*	F: GCACCGTCAAGGCTGAGAAC	138	NM_002046.5
	R: TGGTGAAGACGCCAGTGGA		

*BMI1*, BMI1 proto-oncogene polycomb ring finger; *CDC25A*, cell division cycle 25A; *CDK4*, cyclin-dependent kinase 4; *CDK6*, cyclin-dependent kinase 6; *CYCLIN D*, D-type cyclin; *GSK3B*, glycogen synthase kinase 3 beta; *MYC*, MYC proto-oncogene, bHLH transcription factor; *P15*, cyclin-dependent kinase inhibitor 2B (CDKN2B); *P16*, cyclin-dependent kinase inhibitor 2A (CDKN2A); *P18*, cyclin-dependent kinase inhibitor 2C (CDKN2C); *P19*, cyclin-dependent kinase inhibitor p19INK4d; *RB1*, RB transcriptional corepressor 1; *SMAD3*, SMAD family member 3; *SMAD4*, SMAD family member 4; *GAPDH*, glyceraldehyde-3-phosphate dehydrogenase.

## Data Availability

All relevant data are available in the cited public databases and referenced publications and any inquiries can be directed to R. T. (mail: takeuchi.reiri@nihon-u.ac.jp).

## Supplementary Materials

The following supporting information can be downloaded at https://www.mdpi.com/article/10.3390/diseases12120322/s1. Figure S1. Original Images of Figure 4.
